# The influence of context on the effectiveness of hospital quality improvement strategies: a review of systematic reviews

**DOI:** 10.1186/s12913-015-0906-0

**Published:** 2015-07-22

**Authors:** Dionne S. Kringos, Rosa Sunol, Cordula Wagner, Russell Mannion, Philippe Michel, Niek S. Klazinga, Oliver Groene, on behalf of the DUQuE Consortium

**Affiliations:** Department of Public Health, Academic Medical Center (AMC) - University of Amsterdam, PO Box 22660, 1100 DD Amsterdam, The Netherlands; Avedis Donabedian Research Institute, University Autonomous of Barcelona, C/Provenza 293, Pral. 08037 Barcelona, Spain; NIVEL, Netherlands Institute for Health Services Research, PO Box 1568, 3500 BN Utrecht, The Netherlands; Health Services Management Centre, University of Birmingham, Birmingham, B15 2RT UK; Quality and Safety Department, Lyon University, Hospital Network, Lyon, France; Department of Health Services Research and Policy, Faculty of Public Health and Policy, London School of Hygiene & Tropical Medicine, 15-17 Tavistock Place, London, WC1H 9SH UK; Avedis Donabedian Research Institute (FAD), Universitat Autonoma de Barcelona, ᅟ, Spain; Red de investigación en servicios de salud en enfermedades crónicas REDISSEC, ᅟ, Spain

## Abstract

**Background:**

It is now widely accepted that the mixed effect and success rates of strategies to improve quality and safety in health care are in part due to the different contexts in which the interventions are planned and implemented. The objectives of this study were to (i) describe the reporting of contextual factors in the literature on the effectiveness of quality improvement strategies, (ii) assess the relationship between effectiveness and contextual factors, and (iii) analyse the importance of contextual factors.

**Methods:**

We conducted an umbrella review of systematic reviews searching the following databases: PubMed, Cochrane Database of Systematic Reviews, Embase and CINAHL. The search focused on quality improvement strategies included in the Cochrane Effective Practice and Organisation of Care Group taxonomy. We extracted data on quality improvement effectiveness and context factors. The latter were categorized according to the Model for Understanding Success in Quality tool.

**Results:**

We included 56 systematic reviews in this study of which only 35 described contextual factors related with the effectiveness of quality improvement interventions. The most frequently reported contextual factors were: quality improvement team (*n* = 12), quality improvement support and capacity (*n* = 11), organization (*n* = 9), micro-system (*n* = 8), and external environment (*n* = 4). Overall, context factors were poorly reported. Where they were reported, they seem to explain differences in quality improvement effectiveness; however, publication bias may contribute to the observed differences.

**Conclusions:**

Contextual factors may influence the effectiveness of quality improvement interventions, in particular at the level of the clinical micro-system. Future research on the implementation and effectiveness of quality improvement interventions should emphasize formative evaluation to elicit information on context factors and report on them in a more systematic way in order to better appreciate their relative importance.

**Electronic supplementary material:**

The online version of this article (doi:10.1186/s12913-015-0906-0) contains supplementary material, which is available to authorized users.

## Background

A growing body of research demonstrates the effectiveness of strategies to improve quality and enhance patient safety (QI strategies) [1]. Yet, at the same time the contextual factors affecting the implementation and effectiveness of these strategies are not well understood [2]. Grimshaw et al. provided the first comprehensive review on the effect of interventions to change provider behaviour [3]. This overview of systematic reviews provided invaluable insight into the effectiveness of quality improvement strategies. It raised concerns about the strength of the evidence base and noted that the majority of interventions are effective under some circumstances, although the authors did not systematically explore what such circumstances might be. Scott conducted a similar, albeit less detailed review and concluded that few studies so far have investigated contextual and implementation factors in detail [4]. More recently, Conry et al. conducted yet another review of interventions to improve the quality of care in hospitals and concluded that the lack of theoretically sound research methods that elucidated why interventions work (or do not work) might be a key reason for the slow uptake of the research evidence in health care settings [5].

It is now accepted that the mixed effect and success rates of QI strategies are in part due to the different contexts in which the interventions are planned and implemented [6–8]. An intervention that works in one setting does not necessarily work in another. ‘Context’ for quality improvement has been defined to include those factors that potentially mediate the effect of the intervention, such as leadership, personal skills, organizational resources or data availability [8]. More recently, specific definitions and categorizations of context have been proposed [9, 10]. Often it is neither feasible nor appropriate to adjust for these factors in an analysis of effectiveness. This is, firstly, because it is unlikely that data on all such factors are available and, secondly, because adjustment for context might mask rather than highlight the importance of such factors. As the potential generalisability of findings on the effectiveness of QI strategies (which often include organizational interventions) is much more limited than the generalizability of clinical trials (for example on the pharmacokinetic response to a drug in a defined group of patients), the question ‘does the QI strategy work’ is only of initial interest. The broader question ‘why, when, where, and for whom it works most effectively’ is of much greater concern and practical importance [11]. Having a thorough understanding of the underlying mechanisms that make an intervention work, will allow for successful application of the intervention in other settings and help improving its effectiveness.

The objectives of this paper are therefore to (i) describe the reporting of contextual factors in the literature on the effectiveness of QI strategies, (ii) assess the relationship between these contextual factors and the effectiveness of QI strategies, and (iii) analyze the importance of contextual factors.

## Methods

### Search strategy

We conducted a review of systematic reviews of the literature on the effectiveness of QI strategies [12]. The following electronic databases were searched: PubMed, Cochrane Database of Systematic Reviews, Embase and CINAHL. The search was limited to literature reviews published in English language between January 2000 and November 2012. The search focused on QI strategies included in the Cochrane Effective Practice and Organisation of Care (EPOC) Group taxonomy, which include various forms of continuing medical education (CME), quality assurance projects, financial, organizational, or regulatory interventions that can affect the ability of health care professionals to deliver services more effectively and efficiently [13]. A Boolean search strategy for PubMed was developed (see Additional file 1) covering all quality management topics, including a combination of text words and Medical Subject Headings (MeSH) terms, searched in titles and abstracts of studies. The Boolean search strategy was adapted for the other databases.

### Methods of screening and selection criteria

**Fig. 1:**
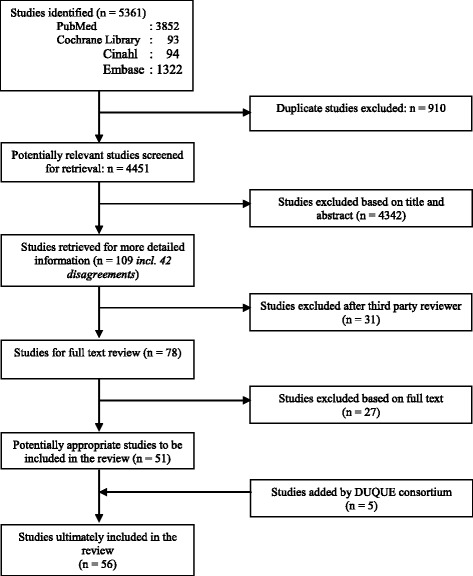
Study selection process. Legend: Figure 1 indicates the study selection process of the systematic literature review

The review strategy was guided by a manual for performing systematic literature reviews on a health services research topic [14]. The results from the databases were checked for duplicates using Reference Manager. This was followed by a three-step screening procedure. Studies were excluded if they did not focus on the effectiveness, performance or impact of quality management strategies in hospital settings. An initial screening of studies was based on titles and abstract, performed by two reviewers (OG, DK) independently. In the second screening, the full texts of the reviews were assessed for inclusion by OG and DK independently. In the third step, the final list of included studies was evaluated for their completeness by a panel of quality management experts from European countries, comprised of mostly senior researchers and medical professionals who participated in a European Commission (EC) funded research project (Deeping our understanding of quality improvement in Europe (DUQuE), see www.duque.eu and acknowledgment section). This evaluation led to five additions to the publication list. Only systematic literature reviews with a focus on the effectiveness, performance or impact of quality management strategies in hospital settings were included. This covered both qualitative and qualitative reviews and meta-analyses. Figure 1 shows the complete study selection process, including the number of disagreements among reviewers which were resolved by a third independent reviewer (RS).

### Data extraction

Data on the effectiveness of quality management strategies and the influence of both internal and external contextual factors were extracted using a structured data entry form. Panel experts from the DUQuE team were each independently assigned to extract the data from a limited set of studies which fit with their expertise. The following information was extracted from the studies that met our inclusion criteria: type of QI intervention, number of included studies and participants, objective, description of intervention, description of primary and secondary outcome, effect of primary and secondary outcome, and contextual factors.

Data extraction on contextual factors was categorized according to an assessment tool based on the Model for Understanding Success in Quality (MUSIQ) (Textbox 1) [9]. We coded context-based factors against the MUSIQ tool and added up the number of studies which displayed each one and noted if the study had produced an effective outcome.

The MUSIQ tool was developed by Kaplan et al. to facilitate research on the contextual factors affecting QI strategies, and has been shown to be reliable and valid [9, 15]. It identifies 25 contextual factors for quality improvement, covering six overarching themes that they labelled as: external environment, organization, quality improvement capacity, the clinical microsystem, the quality improvement team, and a number of miscellaneous issues. Detailed descriptions of the six themes and 25 contextual factors are included in Additional file 2: Table S1.

Textbox 1: Domains of the MUSIQ Tool [9]

**Table:** 

●	*External environment* (external motivators, project sponsorship),
●	*Organization* (QI leadership, senior leader project sponsor, culture supportive of QI, maturity of organizational QI, physician payment structure),
●	*Quality improvement support and capacity* (data infrastructure, resource availability, workforce focus on QI),
●	*Microsystem* (QI leadership, culture supportive of QI, capability for improvement, motivation to change),
●	*Quality improvement team* (team diversity, physician involvement, subject matter expert, team tenure, prior experience with QI, team leadership, team decision-making process, team norms and team QI skill) and
●	*Miscellaneous* (trigger events, task strategic importance to the organization).

All data entry forms were double-checked by two independent reviewers (OG, DK) and complemented or altered when both reviewers agreed. In case of disagreement, a third independent reviewer (RS) made the final decision. The quality of the included studies was assessed using a valid and reliable [16, 17] measurement tool to assess the methodological quality of systematic reviews (AMSTAR) [18]. The overall quality of each study was assessed by the proportion of items of the AMSTAR Checklist each study complied with (number of “Yes” answers to 11 questions). Studies are rated as low quality when AMSTAR score is 0–4, moderate quality when AMSTAR score is 5–8, and high quality when AMSTAR score 9–11 [19]. The quality assessment was undertaken by two independent reviewers (OG, DK).

### Ethics

This research did not involve human subjects. Ethics approval is covered by the DUQUE project, financed by the EU Commission under grant agreement number 241822 and approved by the Health department of the Government of Catalonia, Spain.

## Results

We included 56 systematic reviews in this study (Fig. 1).

The reviews address QI interventions such as health care accreditation (*n* = 3), local leadership (*n* = 1), continuing medical education (*n* = 3), nurturing patient safety cultures (*n* = 2), promoting organizational culture (*n* = 3), computerised clinical decision support systems (*n* = 16), guideline dissemination and implementation (*n* = 2), interventions to improve patient handovers (*n* = 5), patient-centred care interventions (*n* = 3), six sigma and lean continuous quality improvement interventions (*n* = 3), the use of performance information (*n* = 6), audit and feedback (*n* = 2), hospital incident reporting (*n* = 2), safety checklists (*n* = 1), educational outreach visits (*n* = 1), and multi-faceted quality improvement interventions (*n* = 3). Table 1 gives an overview of all studies included, by type of QI intervention, author, year, AMSTAR score, number of studies included (Table 1). In addition, it specifies the countries included and the number of participants. Reviews included between 2 and 235 individual studies, covering mostly North America, Europe and South-East Asia, with very few studies from South America and the African continent. The median AMSTAR score is 7 (moderate quality), but for studies published from 2010 onwards it increased to 8.5 (moderately high quality) (see also Table 1, footnote for comparison of average AMSTAR code by year).

**Table 1:** Study characteristics

Quality improvement strategy	Author(s)	Year	AMSTAR score out of 11 points	Number of studies included	Countries included	Number of participants
Accreditation of health care services	Alkhenizan, Shaw [33]	2011	6	26	South Africa, Zambia, Australia, Denmark, United States, Egypt, Philippines, Japan, Canada.	Hospitals (*N* = 13), Units (*N* = 8), Patients (*N* = 2), Facilities (*N* = 1), Employees (*N* = 1), Program (*N* = 1)
	Flodgren, Pomey, Taber et al. [31]	2011	9	2	England, South Africa	England: all acute hospital trusts. South Africa: 18 hospitals
	Greenfield, Braithwaite [34]	2008	7	66	Ireland, United Kingdom, Australia, France, Italy, Spain, Canada, U.S.	Not specified.
Local leadership	Thomson O’Brien, Oxman, Haynes et al. [27]	2000	6	8	United States, Canada, China	Health professionals (N > 296), US communities (providing care for patients with cancer; *N* = 6), Hospitals (deliveries; *N* = 20), Hong Kong hospital (*N* = 1), Canadian Community hospitals (deliveries; *N* = 16); US hospitals (AMI; *N* = 37), US hospitals (rheumatoid arthritis; *N* = 6), US hospitals (chronic obstructive pulmonary disease; *N* = 16), US hospitals (osteoarthritis; *N* = 6).
Continuing medical education	Bloom [28]	2005	3	26	Not specified	Not specified
	Lam-Antoniades, Ratnapalan, Tait [58]	2009	5	15	Not specified	Nursing facility managers (*N* = 45), Physicians/Nurses (*N* = 2172)
	O’Brien, Freemantle, Oxman et al. [59]	2001	8	32	United States (*N* = 24), United Kingdom (*N* = 2), Australia (*N* = 1), Brazil (*N* = 1), France (*N* = 1), Indonesia (*N* = 1), Sri Lanka (*N* = 1), Zambia (*N* = 1)	2995 health professionals
Promoting a consistent positive patient safety culture across the hospital	Morello, Lowthian, Barker [20]	2013	9	21	United States (*N* = 15), UK (*N* = 3), Canada (*N* = 1), Europe (*N* = 1), Australia (*N* = 1)	Not specified
	Weaver, Lubomski, Wilson et al. [41]	2013	7	33	United States, United Kingdom, Canada. Australia. N not specified by country.	Not specified. Study sample sizes ranged from 5461 persons working in 144 units in a single hospital to 28 individuals working within a single hospital unit.
Promoting a consistent positive organizational culture across the hospital	Griffiths, Renz, Hughes et al. [21]	2009	6	30	Argentina (*N* = 1), Canada (*N* = 2), England (*N* = 6), France (*N* = 1), Malta (*N* = 1), Switzerland (*N* = 1), Thailand (*N* = 1), United Kingdom (*N* = 3), United States (*N* = 5), Not specified (*N* = 9)	Nurse managers (*N* = 91), patients (*N* = 1070), nurses (*N* = 2013), physicians (*N* = 188), managerial staff (*N* = 144), hospitals (*N* = 301), not specified (*N* = 3)
	Parmelli, Flodgren, Beyer et al. [60]	2011	7	2	USA (*N* = 2)	Not specified.
	Scott, Mannion, Marshall et al. [61]	2003	8	10	UK (*N* = 2), United States (*N* = 8), Canada (*N* = 1)	Patients (*N* = 7605), managers (*N* = 77), management teams (*N* = 536), nurses (*N* = 899), physicians (*N* = 2504), not specified (*N* = 1)
Computerised clinical decision support systems	Brand, Barker, Morello et al. [62]	2012	4	57	Not specified.	Not specified.
	Bright, Wong, Dhurjati et al. [63]	2012	10	148	United States (*N* = 189), Europe (*N* = 62), Canada (*N* = 24), Multi-country (*N* = 10), Brazil (*N* = 1), Australia (*N* = 1), New Zealand (*N* = 1), Not specified (*N* = 5)	Not specified.
	Chan, Chan, Cafazzo et al. [22]	2012	10	18	North America (*N* = 17), Europe (*N* = 1)	Patients (*N* = 44529)
	Chaudhry, Wang, Wu et al. [64]	2006	6	257	Not specified.	Not specified.
	Damiani, Pinnarelli, Scopelliti et al. [65]	2009	5	22	United States (*N* = 15), United Kingdom (*N* = 2), Italy (*N* = 1), Switzerland (*N* = 1), Canada (*N* = 1), Australia (*N* = 1), Costa Rica (*N* = 1)	Not specified.
	Damiani, Pinnarelli, Colosimo et al. [49]	2010	8	45	Europe (*N* = 11), United States (*N* = 33), Oceania (*N* = 1)	Inpatient patients (*N* = not specified); outpatient patients (*N* = not specified); physicians (*N* = not specified); other care givers (*N* = not specified).
	Garg, Adhikari, McDonald et al. [23]	2005	9	100	United States (*N* = 69), United Kingdom (*N* = 14), Canada (*N* = 5), Autralia (*N* = 4), Italy (*N* = 2), Austria (*N* = 1), France (*N* = 1), Germany (*N* = 1), Israel (*N* = 1), Norway (*N* = 1), Switzerland (*N* = 1)	Practitioners or practices (N > 3826); patients (N > 92,895).
	Hemens, Holbrook, Tonkin et al. [66]	2011	8	65	United States (*N* = 44), EU/EEA countries (*N* = 13), Canada (*N* = 3), other/multiple countries (*N* = 5)	Health professionals (*N* = 8,932); patients (*N* = 1,246,686)
	Jamal, McKenzie, Clark [67]	2009	7	23	United States (*N* = 14), United Kingdom (*N* = 3), France (*N* = 3), Norway (*N* = 1), The Netherlands (*N* = 1), Canada (*N* = 1)	Health professionals (*N* = not specified)
	Kaushal, Shojania, Bates [29]	2003	7	12	USA (*N* = 12)	Patients (*N* = ranging from 17 to 7490 per study)
	Kawamoto, Houlihan, Balas et al. [68]	2005	7	70	Not specified.	Clinicians (*N* = 6000); patients (*N* = 130,000)
	Main, Moxham, Wyatt et al. [35]	2010	9	24	United States (*N* = 17), United Kingdom (*N* = 2), Spain (*N* = 2), France (*N* = 1), The Netherlands (*N* = 1), Belgium (*N* = 1)	Patients (*N* = 264,405); Physicians (*N* = 2,363)
	Pearson, Moxey, Robertson et al. [30]	2009	7	56	North America (*N* = 39), Europe (*N* = 15), other (*N* = 2)	Health professionals (*N* = not specified)
	Sahota, Lloyd, Ramakrishna et al. [69]	2011	9	36	United States (*N* = 22), The Netherlands (*N* = 4), United Kingdom (*N* = 3), Germany (*N* = 2), New Zealand (*N* = 2), Australia (*N* = 2), Brazil (*N* = 1), Canada (*N* = 1), Denmark (*N* = 1), Israel (*N* = 1), Lithuania (*N* = 1), Norway (*N* = 1), Portugal (*N* = 1). Note: Some of the studies were performed in multiple countries.	Health professionals (*N* = 3,417); patients (*N* = 202,491)
	Shojania, Jennings, Mayhew et al. [70]	2009	11	28	United States (*N* = 19), United Kingdom (*N* = 2), Italy (*N* = 1), Norway (*N* = 1), Australia (*N* = 1), Canada (*N* = 2), New Zealand (*N* = 1), The Netherlands (*N* = 1)	Provider teams (*N* = 10); providers (*N* = 1,138)
	Wong, Yu, Holbrook [71]	2010	6	4	Canada (*N* = 1), Israel (*N* = 1), United States (*N* = 2)	Patient visits (*N* = 80,471)
Guidelines dissemination and implementation	Grimshaw, Eccles, Thomas et al. [72]	2006	9	235	United States (*N* = 167); Other countries (*N* = 68)	Not specified.
	Grimshaw, Thomas, MacLennan [38]	2004	9	235	United States (*N* = 167), United Kingdom (*N* = 26), Canada (*N* = 14), Australia (*N* = 2), The Netherlands (*N* = 1), Denmark (*N* = 1), France (*N* = 1), Germany (*N* = 1), Israel (*N* = 1), Mexico (*N* = 1), New Zealand (*N* = 1), Norway (*N* = 1), Norway (*N* = 1), Sweden (*N* = 1), Thailand (*N* = 1),	Physicians (*N* = not specified)
Interventions to improve handovers	Arora, Manjarrez, Dressler et al. [73]	2009	4	10	Not specified.	Nurses (*N* = 38); patients (*N* = 3843); medical residents (*N* = 107)
	Gordon, Findley [74]	2011	5	10	Not specified.	Health professionals (*N* = 343)
	Mistianen, Francke, Poot [75]	2007	9	15	Not specified.	Patients (*N* = not specified)
	Ong, Coiera [24]	2011	6	24	United Kingdom (*N* = 1); Autralia (*N* = 1); Other (*N* = 22)	Malpractice claims (*N* = 444); handover incidents (*N* = 334); critical incidents (*N* = 176); transfers (*N* = 323); nurses (*N* = 579); clinicians (*N* = 458); patients (*N* = 3974)
	Shepperd, Parkes, McClaran et al. [47]	2004	9	11	United States (*N* = 5), United Kingdom (*N* = 3), Canada (*N* = 2), Denmark (*N* = 1)	Patients (*N* = 5448)
Patient-centred care interventions	Coulter, Ellins [76]	2007	2	129	Not specified.	Not specified.
	Lewin, Skea, Entwistle [77]	2001	8	17	North America (*N* = 11); United Kingdom (*N* = 3); Switzerland (*N* = 1); Norway (*N* = 1); Trinidad and Tobago (*N* = 1)	Health professionals (*N* = not specified); patients (*N* = not specified)
	Stone, Pogorzelska, Kunches et al. [51]	2008	5	42	United States (*N* = 27); Other (*N* = 15)	Patients (*N* = nearly 200,000)
Six sigma and Lean for continuous quality improvement	DelliFraine, Langabeer II, Nembhard [78]	2010	7	34	The Netherlands (*N* = 1); other countries (*N* = 33)	Hospital departments (*N* = 12); hospitals (*N* = 11); managed care company (*N* = 1)
	Glasgow, Scott-Caziewell, Kaboli [32]	2010	8	47	United States (*N* = 45); Australia (*N* = 1); The Netherlands (*N* = 1)	Hospital/department (*N* = 35); Other not specified.
	Nicolay, Purkayastha, Greenhalgh [39]	2012	11	34	United Kingdom (*N* = 1); Switzerland (*N* = 1); India (*N* = 1); Finland (*N* = 1); Australia (*N* = 1); Taiwan (*N* = 2); Germany (*N* = 2); France (*N* = 2); The Netherlands (*N* = 3); United States (*N* = 20)	Patients (*N* = 293,406); hospitals (*N* = 8); Not specified (7 studies).
Performance information	Conry, Humphries, Morgan et al. [5]	2012	9	20	Not specified.	Patients (*N* = 17,622); nurses (*N* = 69); hospital (*N* = 2117); physician (*N* = 23); not specified (2 studies)
	De Vos, Graafmans, Kooistra et al. [40]	2009	5	21	United States (*N* = 17); Canada (*N* = 1); Australia (*N* = 1); Sweden (*N* = 1); Laos (*N* = 1)	Hospitals (*N* = 1988)
	Ketelaar, Faber, Flottorp et al. [52]	2011	9	4	United States (*N* = 3); Canada (*N* = 1)	New medicaid beneficiaries or enrolees (*N* = 24,856); Hospitals (*N* = 86); Not specified (1 study)
	Marshall, Shekelle, Leatherman et al. [79]	2000	2	21	United States (*N* = 21)	Health care providers (*N* = 14); health care consumers (*N* = 3); health care purchasers (*N* = 2); health care providers and purchasers (*N* = 1); hospitals (*N* = 3)
	Schauffler and Mordavsky [25]	2001	0	32	United States (*N* = 32)	Health care consumers (*N* = 14); health care providers (*N* = 14); health care purchasers (*N* = 3); health report cards (*N* = 1), hospitals (*N* = 1)
	Veloski, Boex, Grasberger et al. [36]	2006	4	41	Not specified.	Not specified.
Audit and feedback	Hysong [80]	2009	5	19	Not specified.	Not specified.
	Ivers, Jamtvedt, Flottorp et al. [37]	2012	11	140	United States (*N* = 69); Canada (*N* = 11); UK/Ireland (*N* = 21); Australia/New Zealand (*N* = 10); Sudan (*N* = 2); Thailand (*N* = 1); Laos (*N* = 1); other (*N* = 25).	clusters/groups of health providers (*N* = 2310); health professionals (*N* = 2053)
Hospital incident reporting	Benn, Koutantji, Wallace et al. [26]	2009	6	23	United Kingdom (*N* = 4); United States (*N* = 13); New Zealand/ Australia (*N* = 3); France (*N* = 1); Japan (*N* = 1); Israel (*N* = 1)	Not specified.
	Percarpio, Watts, Weeks [81]	2008	7	38	Not specified.	Not specified.
Safety checklists	Ko, Turner and Finnigan [48]	2011	11	9	Not specified.	Intensive care unit (*N* = 5); emergency department (*N* = 2); Surgery (*N* = 1); Acute Care (*N* = 1)
Educational outreach visits	O’Brien, Rogers, Jamtvedt et al. [82]	2007	10	69	North America (*N* = 23); United Kingdom (*N* = 22); Europe (*N* = 14); Australia (*N* = 8); Indonesia (*N* = 2); Thailand (*N* = 1)	Health professionals (N > 15,000)
Multiple quality improvement strategies	Aboelela, Stone, Larson [50]	2007	10	33	North America (*N* = 17); Europe (*N* = 7); South America (*N* = 5); Middle East/Asia (*N* = 4)	Acute care (general units; *N* = 4); ICU (*N* = 20); entire hospital (*N* = 9)
	Grimshaw, Shirran, Thomas et al. [3]	2001	8	41	Not specified.	Not specified.
	Scott [4]	2009	0	Not specified.	Not specified.	Not specified.

Average AMSTAR score by year (*n* = # studies): *yr 2000*: 4.0 (*n* = 2); *2001:* 6.0 (*n* = 4); *2003:* 7.5 (*n* = 2); *2004:* 9.0 (*n* = 2); *2005:* 6.3 (*n* = 3); *2006:* 6.3 (*n* = 3); *2007:* 7.8 (*n* = 4); *2008:* 6.3 (*n* = 3); *2009:* 5.5 (*n* = 11); *2010:* 7.6 (*n* = 5); *2011:* 7.8 (*n* = 9); *2012:* 9.2 (*n* = 6); *2013:* 8.0 (*n* = 2)

In Additional file 3: Table S2 we summarize the information on contextual factors extracted from the systematic reviews. For each study, we identified the type of contextual factor according to the MUSIQ model and describe how they relate to QI effectiveness (Additional file 3: Table S2). Only 35 of the 56 studies described contextual factors related with the effectiveness of QI interventions. Other studies were exclusively focussed on describing the effectiveness of QI interventions, keeping the contextual factors outside the scope of their study. The most frequently reported contextual factors that were found to be associated with the effectiveness of interventions were the following: external environment (external motivator, *n* = 4), organization (maturity of organizational QI, *n* = 5; QI leadership, *n* = 4), QI support and capacity (resource availability, *n* = 6; data infrastructure, *n* = 5), micro-system (capability for improvement, *n* = 5; culture supportive of QI, *n* = 3), and QI team (physician involvement, *n* = 5; team diversity, *n* = 4; including a subject matter expert, *n* = 3). Contextual factors identified in the MUSIQ tool but not reported in the reviews were QI team norms, task strategic importance to the organization and trigger events. Contextual factors that are not currently included in the MUSIQ tool, but were reported in various studies (*n* = 8) included organizational level of programme implementation, patient turnover and bed occupancy, staffing levels, quality of evidence and guidelines, maturity of systems on which CDSS are based, trust in and quality of information sources and educational outreach visitors [20–27]. In the following section we summarize these contextual factors using the six domains of the MUSIQ model: external environment, organization, quality improvement capacity, the clinical micro-system, the quality improvement team, and miscellaneous issues.

### External environment

In terms of direct effects, financial incentives or administrative support were related with the effectiveness of QI strategies [5, 28]. Resource requirements, too, were a key factor, in particular for large up-front organizational support and capital investment required to introduce a Computerised Physician Order Entry [29]. QI strategies were not equally effective across health care settings: Clinical decision support systems (CDSS) were more effective in institutional settings than community settings due to types of conditions, stricter controls on professional behaviour, and different attitudes of professionals towards externally imposed rules [30]. The external environment also affected the interpretation of QI effectiveness more broadly. Priority areas of external inspections differed between high and low income countries, making the results of studies on the effectiveness of accreditation programmes difficult to compare and transfer [31].

### Organization

There was a substantial amount of literature referring to the effect of supportive organizational cultures [21, 32, 33]. The creation of a patient safety culture (including having clear policies and actively support training) positively impacted on the effectiveness of infection control policies [21], on the implementation of Six Sigma and Lean approaches to QI) [32]. The visible support of managerial staff (both at ward/unit and above ward/unit level) also impacted positively on the effectiveness of accreditation of health care services [34], on clinical decision support systems [35], and on hospital incident reporting [26].

Another example for organizational context factors was the embedding of feedback systems in organizational QI: Feedback on a physician’s clinical performance was more likely to be effective when provided by an authoritative, credible source, systematically over multiple years [36], and a high frequency of performing audit and feedback increased its effectiveness in modifying health professionals’ behaviour [37]. Having a closed safety-feedback cycle (e.g. effective dissemination channels and the capacity for rapid action) at all levels of the organization positively affected the effectiveness of hospital incident reporting [26].

The absence of an effective multidisciplinary infection control team perceived as exercising positive leadership at ward or unit level was a risk for the effectiveness of infection control strategies on health care associated infections [21].

### QI support and capacity

The availability and functionality of information technology (IT) systems facilitated data collection and improved the effectiveness of QI interventions [38], notably, interventions targeting handovers [24], accreditation programs in rural health care services [34], and Six Sigma [39]. Sufficient resources, too, were paramount for the implementation of QI strategies [38, 40, 41]. Insufficient administrative support impacted on the effectiveness of interventions promoting safety cultures [41] or on strategies aimed at implementing quality indicators [40]. An excessive workload (not matched to available staffing) and insufficient staff training were a risk for the effectiveness of Six Sigma [39] and infection control strategies on health care associated infections [21]. High staff turnover and an excessive use of external staff members limited the effectiveness of infection control strategies [21].

### Microsystem

Clinical micro-systems have previously been described as the key settings in which QI interventions are implemented [42–46]. Low staff morale and scepticism of health care professionals towards the positive impact of QI interventions (e.g. accreditation programs or CDSS) on the quality of health care services were serious barriers to the successful implementation of QI interventions [21, 23, 33]. Alignment of physicians’ views on the content and implementation of interventions aimed at improving handovers was beneficial to its effectiveness [24, 47]. The training or education in the proper use of QI strategies (e.g. safety checklists, accreditation standards) was beneficial to the effectiveness of the QI strategies [34, 48]. Integrating QI strategies in the working practices of health professionals promoted the effectiveness of the QI strategies [23] and the ‘motivation to change’ [22, 40].

### QI team

Team composition is a major determinant for QI effectiveness. Involving physicians in the development and implementation of QI interventions (such as CDSS, accreditation programs, audit and feedback) has been shown to be an important success factor for their effectiveness [22, 23, 34, 37, 49]. Involving multidisciplinary QI teams (including nurses and physicians, and sometimes also pharmacists) in the development and implementation of QI strategies increased the effectiveness of the intervention [22, 24, 50, 51]. ‘Subject matter experts’, where more than one team member has detailed knowledge about the outcome, process, or system being changed were shown to be beneficial for the a range of QI strategies (CDSS, CME, Six Sigma and Lean). Awareness, attitude, knowledge of and understanding performance data (generic ‘QI skills’) [52] were all essential facilitators for the implementation of QI interventions.

### Miscellaneous

A number of additional contextual factors were identified from the literature, not all of which are addressed in the MUSIQ tool. These include, in particular, structural factors of service organization, including turnover of staff or bed occupancy [21], workload and time constraints [24], but also the flexibility to update the QI intervention (guidelines or computerised decision support systems) [22, 23]. Detailed information on the contextual factors identified in the literature is presented in Additional file 3.

## Discussion

A number of previous studies have reviewed the effectiveness of quality improvement strategies, but this is to our best knowledge the first study to systematically assess a broad range of associated context factors and their relationship with the effectiveness of multiple quality improvement strategies in health care. This study has shown that, overall, context factors were poorly reported in the current literature. This is a very important finding since those studies that did report it demonstrated substantial differences in QI effectiveness, depending on the presence or absence of contextual factors. Given the heterogeneity of the literature few systematic reviews included in the analysis were able to pursue a quantitative synthesis and stratify this analysis on the context factors identified.

Context factors most frequently reported related to the three MUSIQ domains *microsystem*, *QI support and capacity* or *QI team*. This aligns with recent analysis of the concepts underlying the MUSIQ tool, which identified that these domains exhibit significant effects on QI performance outcomes [15]. Of significance for improvement efforts these domains are also most amenable to local adjustment. We also identified context factors that are not currently included in the MUSIQ tool, such as those relating to structural characteristics or the heterogeneity observed in the relationship between context and multiple outcome measures [20–27]. Since the publication of the MUSIQ tool several other key publications on the role of context have emerged [2, 10, 15, 53]. Our findings might help to inform these conceptual developments. Moreover, our data may lead to reflections on the broad conceptual nature of existing context models (which emphasize multi-level structures, the role of the external environment, the organization at large and clinical practice), while it seems that all three key domains emerging from this review broadly relate to the clinical microsystem.

### Implications of the study

This study has important implications for future research on the relationship between QI context and effectiveness. For some QI interventions the evidence is substantial, and is supported by clear recommendation on how context factors mediate effectiveness (e.g. the Cochrane review on audit and feedback [37] gives clear indication on the factors and the magnitude to which they increase or attenuate the effect of QI strategies). For other QI interventions, the evidence base is still weak and adaptation and implementation should be pursued with caution, since generalisability is limited when context factors are unknown.

Future studies on the effectiveness of QI interventions need to place more emphasis on studying and reporting outcomes in relation to contextual factors [53]. Pooled averages are misleading and do not reflect the varying contexts in which QI are implemented. Ideally, alongside major evaluations of effectiveness, studies should be conducted looking at context factors and recording such factors using existing tools (e.g. [9, 53]). This would permit future reviews on the effectiveness of QI to stratify on the type of context, sample size permitting. In parallel, qualitative studies (including ethnographic studies) could be conducted to gain a deeper understanding of professional, organizational, cultural and structural context factors [54]. This would be a complex and difficult undertaking. For example, a true investigation of context factors cannot be achieved retrospectively at the stage of writing up a research project, but rather requires engaging multiple perspectives and stakeholders from the start to ensure all relevant aspects of QI implementation (both formal and informal) are considered. Once identified, such factors need to be observed and monitored over time and statistical models could aim to assess the association of context factors with the effectiveness of QI interventions, where appropriate. We have recently illustrated some of the methodological challenges related to these tasks [55, 56] and at current, the multiple relationships and pathways between exposure, outcome, and context variables in research on QI strategies are not yet sufficiently understood. Alternatively, context might be considered as an integral component of the subject area that evolves, changes and interacts with the intervention during the time period of QI project implementation. In this case, in-depth qualitative assessment is needed. Finally, the research output should report on the role of context factors in order to facilitate generalisability and replicability of the QI intervention. This issue, too, has been subject to debate and the Standards for Quality Improvement Reporting Excellence (SQUIRE) guidelines are one approach to address a better understanding of QI context factors [57]. The SQUIRE guidelines demonstrate the difficulties and practical issues when reporting on the factors that potentially impact on a QI intervention. Nevertheless, improving understanding, conceptualization, analysis and reporting of context factors in QI is important to advance the field of research. It will help in understanding the mixed results of some QI interventions and help replicate successful projects or, equally important, inform implementers were replication is unlikely to be successful due to different contexts. Our findings suggest that some of the most relevant context factors are those close to the clinical microsystem in which the QI intervention is delivered. This provides cues for action for improvement practitioners who may include an assessment of such factors, and dedicated change processes, in their local plans for quality improvement.

### Limitations

This study has a number of limitations. The main limitation is that the field of research addressing the role of contextual factors in QI is still developing and currently there is no clear consensus on how to define or assess context factors, which has implications for the reviews included in our umbrella review. While we were able to apply the MUSIQ tool to categorize context factors and facilitate data extraction from the literature, the lack of clear search terms may mean that we might have missed reviews for our study. Moreover, it is unclear how contextual factors were assessed in the original studies included in our list of systematic reviews. This may potentially induce publication bias as positive associations are more likely to have been reported. A meta-regression analysis of the effect of context factors, adjusting for publication bias, would be desirable, but given the heterogeneous reporting of the findings in the literature this was not possible at present. We intentionally searched only for the literature that *primarily* addressed the effectiveness of QI strategies (and not for literature that primarily addressed context factors), as our key interest was the effect of QI and how it is affected by context. In comparison, much of the context literature does not report specifically on QI effectiveness and includes also largely qualitative and mixed methods research. An assessment of this literature would have been beyond the scope of this paper. While acknowledging these limitations, the findings of this review are nevertheless important for an advancement of the understanding of how context factors shape the effectiveness of quality improvement interventions.

## Conclusions

Contextual factors may influence the effectiveness of quality improvement interventions, in particular at the level of the clinical microsystem. Future research on the implementation and effectiveness of QI interventions should emphasize formative evaluation to elicit information on context factors and report on them in a more systematic way in order to better appreciate their relative importance.

## Additional files

Additional file 1:
**Search strategy.** The Boolean search strategy which was developed for PubMed. It covers all quality management topics, including a combination of text words and Medical Subject Headings (MeSH) terms, searched in titles and abstracts of studies. The Boolean search strategy was adapted for the other databases. Additional file 2: Table S1. Contextual factors included in the Model for Understanding Success in Quality (MUSIQ). Detailed descriptions of the six themes and 25 contextual factors that are included in the Model for Understanding Success in Quality (MUSIQ). Additional file 3: Table S2. Context factors and their impact on the effectiveness of quality improvement strategies. Summary of the information on contextual factors extracted from the systematic reviews. For each study, we identified the type of contextual factor according to the MUSIQ model and describe how they relate to QI effectiveness.

## Abbreviations

AMSTAR: A measurement tool to assess the methodological quality of systematic reviews
CDSS: Clinical decision support systems
CME: Continuing medical education
DUQuE: Deeping our understanding of quality improvement in Europe
EC: European commission
EPOC: Group taxonomy: Cochrane effective practice and organisation of care group taxonomy
MeSH: Medical subject headings
MUSIQ: Model for understanding success in quality
QI: Quality improvement
QI strategies: Strategies to improve quality and enhance patient safety
SQUIRE guidelines: Standards for quality improvement reporting excellence guidelines
