# Supplementary material for: The influence of context on the effectiveness of hospital quality improvement strategies: a review of systematic reviews
Source: BMC Health Serv Res. 2015 Jul 22;15:277. doi: 10.1186/s12913-015-0906-0 (PMC4508989; doi:10.1186/s12913-015-0906-0)
Supplement: Additional file 1: — Search strategy. The Boolean search strategy which was developed for PubMed. It covers all quality management topics, including a combination of text words and Medical Subject Headings (MeSH) terms, searched in titles and abstracts of studies. The Boolean search strategy was adapted for the other databases. [file 12913_2015_906_MOESM1_ESM.docx]

**Additional file 1: Search Strategy**

(((((((("2000/01/01"[Date - Publication] : "2012/11/27"[Date - Publication])) AND "english"[Language])) AND (((((systematic literature review*[Title/Abstract]) OR literature review*[Title/Abstract]) OR systematic review*[Title/Abstract]) OR literature search*[Title/Abstract]) OR "review"[Publication Type])) AND ((((((((hospital*[Title/Abstract]) OR department*[Title/Abstract]) OR care[Title/Abstract]) OR ward[Title/Abstract]) OR provider*[Title/Abstract]) OR clinician*[Title/Abstract]) OR physician*[Title/Abstract]) OR nurs*[Title/Abstract])) AND ((((effect*[Title/Abstract]) OR effectiveness[Title/Abstract]) OR performance[Title/Abstract]) OR impact*[Title/Abstract]))) AND ((((((((((((((((((((((quality improvement*[Title/Abstract]) OR improvement strateg*[Title/Abstract]) OR quality polic*[Title/Abstract]) OR quality plan*[Title/Abstract]) OR quality program*[Title/Abstract]) OR annual program*[Title/Abstract]) OR score card*[Title/Abstract]) OR quality report*[Title/Abstract]) OR patient safety[Title/Abstract]) OR clinical governance[Title/Abstract]) OR quality leadership[Title/Abstract]) OR quality assurance[Title/Abstract]) OR quality management[Title/Abstract]) OR quality system[Title/Abstract])) OR ((((((((Patient satisfaction survey*[Title/Abstract]) OR patient survey*[Title/Abstract]) OR staff satisfaction survey*[Title/Abstract]) OR staff survey*[Title/Abstract]) OR satisfaction survey*[Title/Abstract]) OR accountability[Title/Abstract]) OR patient experience* AND survey[Title/Abstract]) OR staff experience* AND survey[Title/Abstract])) OR ((((((((((((((((((((((quality resource*[Title/Abstract]) OR steering group*[Title/Abstract]) OR quality committee*[Title/Abstract]) OR quality officer*[Title/Abstract]) OR quality coordinator*[Title/Abstract]) OR safety officer*[Title/Abstract]) OR safety coordinator*[Title/Abstract]) OR reward*[Title/Abstract]) OR incentive*[Title/Abstract]) OR adverse event report*[Title/Abstract]) OR incident reporting[Title/Abstract]) OR licens*[Title/Abstract]) OR teamwork[Title/Abstract]) OR postgraduate training[Title/Abstract]) OR medical education[Title/Abstract]) OR peer review[Title/Abstract]) OR electronic medical record*[Title/Abstract]) OR electronic patient record*[Title/Abstract]) OR electronic health record*[Title/Abstract]) OR information technology[Title/Abstract]) OR decision support system*[Title/Abstract]) OR computer provider order entry[Title/Abstract])) OR ((((((((guideline compliance[Title/Abstract]) OR audit*[Title/Abstract]) OR complication regist*[Title/Abstract]) OR clinical indicator*[Title/Abstract]) OR performance indicator*[Title/Abstract]) OR performance data[Title/Abstract]) OR performance information[Title/Abstract]) OR accountability[Title/Abstract])) OR ((((external assessment*[Title/Abstract]) OR accreditation*[Title/Abstract]) OR certification*[Title/Abstract]) OR inspection[Title/Abstract])) OR ((Root cause analysis[Title/Abstract]) OR risk management[Title/Abstract])) OR ((((((((((((((((((((((central line infection[Title/Abstract]) AND protocol[Title/Abstract])) OR ((central line infection[Title/Abstract]) AND guideline[Title/Abstract])) OR ((central line infection[Title/Abstract]) AND guideline[Title/Abstract])) OR ((surgical site infection[Title/Abstract]) AND protocol[Title/Abstract])) OR ((surgical site infection[Title/Abstract]) AND guideline[Title/Abstract])) OR ((hospital acquired infection[Title/Abstract]) AND protocol[Title/Abstract])) OR ((hospital acquired infection[Title/Abstract]) AND guideline[Title/Abstract])) OR ((ventilator associated pneumonia[Title/Abstract]) AND protocol[Title/Abstract])) OR ((ventilator associated pneumonia[Title/Abstract]) AND guideline[Title/Abstract])) OR ((pressure ulcer[Title/Abstract]) AND protocol[Title/Abstract])) OR ((pressure ulcer[Title/Abstract]) AND guideline[Title/Abstract])) OR ((falls[Title/Abstract]) AND protocol[Title/Abstract])) OR ((falls[Title/Abstract]) AND guideline[Title/Abstract])) OR ((medication error[Title/Abstract]) AND protocol[Title/Abstract])) OR ((medication error[Title/Abstract]) AND guideline[Title/Abstract])) OR ((medical waste[Title/Abstract]) AND protocol[Title/Abstract])) OR ((medical waste[Title/Abstract]) AND guideline[Title/Abstract])) OR ((handover[Title/Abstract]) AND protocol[Title/Abstract])) OR ((handover[Title/Abstract]) AND guideline[Title/Abstract])) OR (guideline adherence[Title/Abstract]))) OR ((((((((benchmark*[Title/Abstract]) OR adverse event analysis[Title/Abstract]) OR patient record review[Title/Abstract]) OR medical record review[Title/Abstract]) OR care pathway[Title/Abstract]) OR client council[Title/Abstract]) OR complaint* AND analysis[Title/Abstract]) OR patient complaints[Title/Abstract]))
