# Supplementary material for: The influence of context on the effectiveness of hospital quality improvement strategies: a review of systematic reviews
Source: BMC Health Serv Res. 2015 Jul 22;15:277. doi: 10.1186/s12913-015-0906-0 (PMC4508989; doi:10.1186/s12913-015-0906-0)
Supplement: Additional file 2: Table S1. — Contextual factors included in the Model for Understanding Success in Quality (MUSIQ). Detailed descriptions of the six themes and 25 contextual factors that are included in the Model for Understanding Success in Quality (MUSIQ). [file 12913_2015_906_MOESM2_ESM.docx]

**Additional file 2: Table S1**

Contextual factors included in the Model for Understanding Success in Quality (MUSIQ)

| Contextual factor |  | Final definition |
| --- | --- | --- |
| **External environment** | | |
| External motivators |  | Environmental pressures and incentives that stimulate the organisation to improve its performance and quality in the area of focus of this QI project |
| Project sponsorship |  | Substantial and meaningful contributions of personnel, expertise, money, equipment, facilities, or other important resources from outside entities (external to the organisation) with formal relationships with this QI project |
| **Organisation** | | |
| QI leadership |  | Senior management's (CEO, COO, CMO, Senior VP, Board of Directors) governance—guidance, support, oversight, and direction setting—of improvement efforts |
| Senior leader project sponsor |  | Senior leader commitment to champion and support this QI project |
| Culture supportive of QI |  | Values, beliefs, and norms of an organisation that shape the behaviours of staff in pursuing QI |
| Maturity of organisational QI |  | Sophistication of the organisation's QI programme |
| Physician payment structure |  | Physicians are employed and compensated by the organisation |
| **QI support and capacity** | | |
| Data infrastructure |  | Extent to which a system exists to collect, manage, and facilitate the use of data needed to support performance improvement |
| Resource availability |  | Degree to which financial support for QI, including allocation of resources and staff time, is provided |
| Workforce focus on QI |  | Degree to which the organisation develops the workforce through training and engages them in QI through reward systems and expectation setting |
| **Microsystem** | | |
| QI leadership |  | Microsystem leadership capacity for improvement and degree to which they are personally involved in supporting and facilitating improvement efforts |
| Culture supportive of QI |  | Values, beliefs, and norms present in the microsystem that emphasise teamwork, communication, freedom to make decisions, and commitment to improve |
| Capability for improvement |  | Microsystem staff's ability to use QI methods for change |
| Motivation to change |  | Extent to which microsystem staff members have a desire and willingness to improve performance in this area of focus |
| **QI team** | | |
| Team diversity |  | Diversity of team members with respect to professional discipline, personality, motivation, and perspective |
| Physician involvement |  | Contribution of physicians to the QI team efforts |
| Subject matter expert |  | One or more team members is knowledgeable about the outcome, process, or system being changed |
| Team tenure |  | Team members have worked together as a team before |
| Prior QI experience |  | Prior experience with QI |
| Team leadership |  | Team leader's ability to accomplish the goals of the improvement project through guiding the actions of the QI team |
| Team decision-making process |  | Team engages in well designed decision-making practices |
| Team norms |  | Team establishes strong norms of behaviour related to how work is to be carried out and how goals are to be achieved |
| Team QI skill |  | Team's ability to use improvement methods to make changes |
| **Miscellaneous** | | |
| Trigger |  | Presence of a specific event (positive or negative) that stimulates a new emphasis on improving quality in the area of focus of a given QI project |
| Task strategic importance to the organisation |  | Work perceived as part of the organisation's strategic goals |

CEO, chief executive officer; CMO, chief marketing officer; COO, chief operating officer; QI, quality improvement; VP, vice president.
