# Supplementary material for: The influence of context on the effectiveness of hospital quality improvement strategies: a review of systematic reviews
Source: BMC Health Serv Res. 2015 Jul 22;15:277. doi: 10.1186/s12913-015-0906-0 (PMC4508989; doi:10.1186/s12913-015-0906-0)
Supplement: Additional file 3: Table S2. — Context factors and their impact on the effectiveness of quality improvement strategies. Summary of the information on contextual factors extracted from the systematic reviews. For each study, we identified the type of contextual factor according to the MUSIQ model and describe how they relate to QI effectiveness. [file 12913_2015_906_MOESM3_ESM.docx]

**Additional file 3: Table S2**

Context factors and their impact on the effectiveness of quality improvement strategies

| **Accreditation of health care services** | | |
| --- | --- | --- |
| *Author(s)* | *Context factor* | *Impact on effectiveness* |
| Alkhenizan, Shaw [33] | Microsystem: culture supportive of QI | Scepticism of healthcare professionals, and physicians in particular about the positive impact of accreditation programs on the quality of health services is an important barrier to the implementation of accreditation programs. |
| Flodgren, Pomey, Taber et al. [31] | External environment: external motivators | External inspections have a different focus in high income countries, compared to low income countries, making effectiveness studies in different contexts unsuitable for comparison of outcomes. Main aims in high income countries include: evaluation and improvement of safety, clinical effectiveness, consumer information, staff development; while in low income countries, they include: establishing basic facilities and information, and improving access to healthcare services. |
| Greenfield, Braithwaite [34] | QI support and capacity: resource availability, data infrastructure; Microsystem: capability for improvement.  Organization: QI leadership  QI team: physician involvement | Reported causes of failing participation by professional from rural health services to accreditation programs include: cost, difficulty in meeting standards and collecting data.  The process of preparation and undergoing accreditation has been shown to promote change in health organizations. Managers being perceived as participative, having more years of experience, have written more self-studies, and whose faculty support the accreditation process, were likely to have more positive accreditation outcomes.  Involving physicians in the development of clinical indicators along with regular feedback, results in their extensive use and many actions dedicated to improve patient care. |
| **Local opinion leaders** | | |
| *Author(s)* | *Context factor* | *Impact on effectiveness* |
| O’Brien, Oxman, Haynes et al. [27] | Miscellaneous | Local opinion leaders appear to be more effective when their interventions are combined with other complementary interventions, such as reminders, audit and feedback, outreach visits, marking strategies, local consensus processes and patient medicated interventions. |
| **Continuing medical education** | | |
| *Author(s)* | *Context factor* | *Impact on effectiveness* |
| Bloom [28] | External environment: External motivators. | Economic incentives are the best motivator of patient behaviour change. |
| Lam-Antoniades, Ratnapalan, Tait [58] | Not reported |  |
| O’Brien, Freemantle, Oxman et al. [59] | QI team: subject matter expert.  Microsystem: Capability for improvement | Determining the knowledge, attitudes or skills of patients prior to the educational meeting intervention, facilitates change since patient expectations are a barrier to change.  The effect score of CME tends to increase as the complexity of targeted behaviour decreases. |
| **Promoting a consistent positive patient safety culture across the hospital** | | |
| *Author(s)* | *Context factor* | *Impact on effectiveness* |
| Morello, Lowthian, Barker [20] | Miscellaneous: organizational level | There is some evidence that multi-faceted unit-based (as opposed to organizational based) programmes facilitate the effect on patient safety climate and patient outcomes. |
| Weaver, Lubomski, Wilson et al. [41] | QI support and capacity : Resources availability | Administrative support is associated with increased compliance |
| **Promoting a consistent positive organizational culture across the hospital** | | |
| *Author(s)* | *Context factor* | *Impact on effectiveness* |
| Griffiths, Renz, Hughes et al. [21] | Organization: QI leadership  Organization: culture supportive of QI  Organization : Maturity of organizational QI  QI support and capacity : Workforce focus on QI  QI support and capacity : Resources availability  Microsystem: Culture supportive of QI  Miscellaneous | The following factors are a risk for the effectiveness of infection control strategies on health care associated infections:  Weak or negative clinical leadership at ward level and above ward level; absence of clear lines of clinical management and responsibility; excessive span of control among clinical leaders; unclear roles and responsibilities for infection control.  Lack of clear policies and active support for training.  Absence of an effective multidisciplinary infection control team perceived as exercising positive leadership at ward or unit level.  High staff turnover and high use of bank or agency staff.  Workload not matched to available staffing.  Low staff morale.  High patient turnover; high bed occupancy. |
| Parmelli, Flodgren, Beyer et al. [60] | Not reported |  |
| Scott, Mannion, Marshall et al. [61] | Not reported |  |
| **Computerised clinical decision support systems** | | |
| *Author(s)* | *Context factor* | *Impact on effectiveness* |
| Brand, Barker, Morello et al. [62] | Not reported |  |
| Bright, Wong, Dhurjati et al. [63] | Not reported |  |
| Chan, Chan, Cafazzo et al. [22] | Microsystem: motivation to change.  QI team: team diversity  QI team: physician involvement    Miscellaneous | A lack of motivation from physicians to use order sets is a big challenge for its effectiveness. Key factors that can affect physicians’ adoption of the tool are: the quality of the order set, efficiency in using it, awareness of it, and accessibility of the order set.  The formation of a multidisciplinary team (incl. at least physicians, nurses and pharmacists) when developing and implementing an order set increases the willingness of providers to use the order set.  The willingness of physicians to support the order set increases if their peers are involved in the development and implementation process. The establishment of a feedback system is also conducive to the implementation and effectiveness of order sets.  The evidence and guidelines on which order sets are based, should regularly be evaluated and updated to include the most current evidence and/or guidelines. |
| Chaudhry, Wang, Wu et al. [64] | Not reported |  |
| Damiani, Pinnarelli, Scopelliti et al. [65] | Microsystem: Culture supportive of QI | Reactive safety processes can only be implemented through a systematic approach in a culture that accepts a degree of human fallibility and in which blame is reserved for clear cases of negligence, recklessness or criminality. |
| Damiani, Pinnarelli, Colosimo et al. [49] | QI team: physician involvement | The improvement of adherence of physicians to guidelines is likely related to physicians’ involvement in decisions regarding clinical recommendations. |
| Garg, Adhikari, McDonald et al. [23] | Organization: Senior leader project sponsor  Microsystem: capability for improvement  QI team: Physician involvement / subject matter expert  Miscellaneous | Home grown CDSS, and “local champions” are an important success factor.  Barriers to implementation of CDSS, and thus its success, include: failure of practitioner  s to use the CDSS, poor usability or integration into practitioner workflow, or practitioner nonacceptance of computer recommendations.  Better performance was identified in studies in which the trial authors also developed the CDSS software. There is an important motivational effect of a developer’s enthusiasm, creating a more usable and integrated software, better access to technical support and training etc. Compatibility with legacy applications, system maturity, and upgrade availability are important issues facilitating the effectiveness of the CDSS. |
| Hemens, Holbrook, Tonkin et al. [66] | QI support and capacity: Data infrastructure | In contrast to expectations, it was found that CCDSS failure was associated with integration of CCDSS with EMRs and in use in an academic setting. |
| Jamal, McKenzie, Clark [67] | Not reported |  |
| Kaushal, Shojania, Bates [29] | External environment: project sponsorship | Financial and organizational support should be gathered by a health care organization before introducing Computerised Physician Order Entry with CDSSs. CPOE requires large up-front capital investment with more remote, albeit substantial, returns. |
| Kawamoto, Houlihan, Balas et al. [68] | Not reported |  |
| Main, Moxham, Wyatt et al. [35] | Organization: QI leadership | Support for CDSS by management and staff, and having people with the specialist knowledge and skills who are able to develop systems are likely to have a beneficial effect upon clinical practice. |
| Pearson, Moxey, Robertson et al. [30] | External environment: external motivators | Institutional interventions are more effective than those conducted in community practice, because of the type of conditions managed in this setting; the stricter controls on the practices of health care professionals in institutional settings; and a potentially greater willingness to abide by externally imposed rules and management suggestions. |
| Sahota, Lloyd, Ramakrishna et al. [69] | Not reported |  |
| Shojania, Jennings, Mayhew et al. [70] | Not reported |  |
| Wong, Yu, Holbrook [71] | Not reported |  |
| **Guidelines dissemination and implementation** | | |
| *Author(s)* | *Context factor* | *Impact on effectiveness* |
| Grimshaw, Eccles, Thomas et al. [72] | Not reported |  |
| Grimshaw, Thomas, MacLennan [38] | QI support and availability: resource availability; data infrastructure | The availability of sufficient financial resources are an important facilitator for comprehensive guideline dissemination and implementation strategies.  The poor availability and functionality of current IT systems are a barrier for the delivery of interventions through computerised systems. |
| **Interventions to improve handovers** | | |
| *Author(s)* | *Context factor* | *Impact on effectiveness* |
| Arora, Manjarrez, Dressler et al. [73] | Not reported |  |
| Gordon, Findley [74] | QI team: team tenure | Fostering joint professional responsibility and teamwork with regard to error prevention may improve patient safety. |
| Mistianen, Francke, Poot [75] | Not reported |  |
| Ong, Coiera [24] | Organization: maturity of organizational QI  QI team: team diversity  QI team: team decision-making process  Microsystem: culture supportive of QI  QI support and capacity: Data infrastructure  Miscellaneous | Regarding transportation of critically ill patients: failure to communicate arrival time and resources required result in delayed and inadeauate care, and therefore requires formalization of the pretransport coordination process. Time constraints impede handoff communication during the discharge planning process, and therefore require the formalization of handoff to allow for uninterrupted time to conduct handoff. Standardization of handoff content and structure is required to avoid that informal and unstructured handoffs the lead to inadequate information transfer.  Regarding discharge of patients from critical care to specialty ward: ward nurses may lack expertise or confidence in handling critical care patients from the ICU., and therefore requires the introduction of an ICU liaison nurse role to facilitate transfer, providing both coordination and clinical support.  The involvement of all members of a multidisciplinary team in handoff will improve the quality of surgical handoff.  The alignment of physicians’ view for referrals handoff communication across medical specialties through education and hospital wide guidelines avoid incomplete handoff and interprofessional misunderstanding.  Implementation of a centralized information repository to ease access to patient information will avoid difficulties in assessing information and communicating with providers across units or different shifts.  Increasing staffing levels will avoid high workload, time constraint and overcrowding at ED which impede handoff communication. |
| Shepperd, Parkes, McClaran et al. [47] | Microsystem: culture supportive of QI | Professionals having different perceptions of alternative care settings, and funding arrangements, may influence timely discharge. |
| **Patient-centred care interventions** | | |
| *Author(s)* | *Context factor* | *Impact on effectiveness* |
| Coulter, Ellins [76] | QI team: prior QI experience | Ongoing support of health professionals to patients, as they take on new roles, will become increasingly important. To support this, health professionals must be given the opportunity to develop their competencies in patient-centred care and require resources to work collaboratively with patients to help them access and understand health information and to offer support in decision-making. |
| Lewin, Skea, Entwistle [77] | Not reported |  |
| Stone, Pogorzelska, Kunches et al. [51] | QI team: team diversity | Interdisciplinary team work and consistent training, combined with adequate nurse staffing reduce the risk of hospital acquired infections. |
| **Six sigma and Lean for continuous quality improvement** | | |
| *Author(s)* | *Context factor* | *Impact on effectiveness* |
| DelliFraine, Langabeer II, Nembhard [78] | QI support and capacity: workforce focus on QI | One study reported that a lack of participant support hampered the implementation of Six Sigma and Lean. |
| Glasgow, Scott-Caziewell, Kaboli [32] | Organization: culture supportive of QI  QI team: subject matter expert | The creation of a patient safety culture will often be needed to overcome the initial resistance that Six Sigma and Lean often face from frontline staff and managers.  A common first step of successful organizations using Six Sigma and Lean is to gather a group of leaders throughout the organization and to have them observe the process in action at a highly successful organization. The next step is the develop personnel with experiences and expertise by using outside consultants on the short term, and developing full-time in-house talent on the long term, who can support the projects and create acceptance. It is also important to train a critical mass of employees with essential knowledge and experience. |
| Nicolay, Purkayastha, Greenhalgh [39] | QI support and capacity: data infrastructure; resource availability | In particular, Six Sigma requires considerable data collection, staff training in the application of the methodology and in statistical analysis, to be implemented effectively. |
| **Performance information** | | |
| *Author(s)* | *Context factor* | *Impact on effectiveness* |
| Conry, Humphries, Morgan et al. [5] | External environment: external motivators | Lack of administrative support forms a barrier for implementing interventions aimed to improve teamwork. Only when staff are empowered, quality improvements have been reported. |
| De Vos, Graafmans, Kooistra et al. [40] | Microsystem: motivation to change  QI team: team leadership  QI support and capacity: resource availability | Lack of convincement of individual health care professionals of the evidence hampers the effectiveness of the QI intervention.  Another barrier is the lack of mutual accountability and control (no leadership).  Lack of resources (such as time investment and lack of administrative support are other hampering factors. |
| Ketelaar, Faber, Flottorp et al. [52] | QI team: Team QI skill | The effect of performance data is higher for people who understand the provided information. In addition to awareness, attitude, and knowledge of performance data, understanding the data is an essential facilitator for realising changes in behaviour of healthcare consumers, providers or purchasers of care. |
| Marshall, Shekelle, Leatherman et al. [79] | Not reported |  |
| Schauffler and Mordavsky [25] | Miscellaneous | Important barriers for consumers to use consumer report cards is distrust in the source of information, and a lack of understanding and value of the content and use/usability of the published information. |
| Veloski, Boex, Grasberger et al. [36] | Organization: Maturity of organizational QI | Feedback on physicians’ clinical performance is more likely to be effective when provided by an authoritative credible source systematically over multiple years. |
| **Audit and feedback** | | |
| *Author(s)* | *Context factor* | *Impact on effectiveness* |
| Hysong [80] | Not reported |  |
| Ivers, Jamtvedt, Flottorp et al. [37] | Organization: Maturity if organizational QI  QI team: physician involvement | Frequent (monthly) performing audit and feedback increases its effectiveness on changing health professionals’ behaviour.  Audit and feedback is more effective when the responsible person is a supervisor or colleague. |
| **Hospital incident reporting** | | |
| *Author(s)* | *Context factor* | *Impact on effectiveness* |
| Benn, Koutantji, Wallace et al. [26] | Organization: QI leadership  Organization: maturity of organizational QI  Microsystem: capability for improvement  Miscellaneous | Visible senior-level support is essential.  Effective dissemination channels and the capacity for rapid action and need for feedback at all levels of the organization are important facilitators for the effectiveness of incident reporting. A closed safety-feedback cycle that operates as a continuous process is required.  Empowering front-line staff to take responsibility for improving safety in local work systems facilitates improvement.  The information needs to be credible and come from a reliable source.. |
| Percarpio, Watts, Weeks [81] | Not reported |  |
| **Safety checklists** | | |
| *Author(s)* | *Context factor* | *Impact on effectiveness* |
| Ko, Turner and Finnigan [48] | Microsystem: capability for improvement | All studies that showed the effectiveness of safety checklists used some sort of training or education to increase compliance and proper use of the checklists. |
| **Educational outreach visits** | | |
| *Author(s)* | *Context factor* | *Impact on effectiveness* |
| O’Brien, Rogers, Jamtvedt et al. [82] | Not reported (Miscellaneous) | The qualification of the visitor delivering the educational outreach visit is likely to be important for the effectiveness. However, this potential influence has not been studied to date. |
| **Multiple quality improvement strategies** | | |
| *Author(s)* | *Context factor* | *Impact on effectiveness* |
| Aboelela, Stone, Larson [50] | QI team: team diversity | Having multidisciplinary QI teams in place most likely contributes to the effectiveness of behavioural interventions to reduce health care associated infections. |
| Grimshaw, Shirran, Thomas et al. [3] | Not reported |  |
| Scott [4] | Not reported |  |

**References**3. Grimshaw JM, Shirran L, Thomas R, Mowatt G, Fraser C, Bero L, Grilli R, Harvey E, Oxman A, O’Brien MA: **Changing provider behavior: an overview of systematic reviews of interventions*.*** *Med Care* 2001, **39**(Suppl 2):Ii2-45.

4. Scott I: **What are the most effective strategies for improving quality and safety of health care?** *Intern Med J* 2009;**39**:389-400.

5. Conry MC, Humphries N, Morgan K, McGowan Y, Montgomery A, Vedhara K, Panagopoulou E, Mc Gee H: **A 10 year (2000-2010) systematic review of interventions to improve quality of care in hospitals.** *BMC Health Serv Res* 2012;**12**:275.

20. Morello RT, Lowthian JA, Barker AL, McGinnes R, Dunt D, Brand C: **Strategies for improving patient safety culture in hospitals: a systematic review**. *BMJ Qual Saf* 2013;**22**:11-18.

21. Griffiths P, Renz A, Hughes J, Rafferty AM: **Impact of organisation and management factors on infection control in hospitals: a scoping review**. *J Hosp Infect* 2009;**73**:1-14.

22. Chan AJ, Chan J, Cafazzo JA, Rossos PG, Tripp T, Shojania K, Khan T, Easty AC: **Order sets in health care: a systematic review of their effects**. *Int J Technol Assess Health Care* 2012;**28**:235-240.

23. Garg AX, Adhikari NK, McDonald H, Rosas-Arellano MP, Devereaux PJ, Beyene J, Sam J, Haynes RB: **Effects of computerized clinical decision support systems on practitioner performance and patient outcomes: a systematic review**. *JAMA* 2005;**293**:1223-1238.

24. Ong MS, Coiera E: **A systematic review of failures in handoff communication during intrahospital transfers**. *Jt Comm J Qual Patient Saf* 2011;**37**:274-284.

25. Schauffler HH, Mordavsky JK: Consumer reports in health care: do they make a difference? Annu Rev Public Health 2001;22:69-89.

26. Benn J, Koutantji M, Wallace L, Spurgeon P, Rejman M, Healey A, Vincent C: **Feedback from incident reporting: information and action to improve patient safety**. *Qual Saf Health Care* 2009;**18**:11-21.

27. Thomson O'Brien MA, Oxman AD, Haynes RB, Davis DA, Freemantle N, Harvey EL: **Local opinion leaders: effects on professional practice and health care outcomes**. *Cochrane Database Syst Rev* 2000;**2**:Cd000125.

28. Bloom BS: **Effects of continuing medical education on improving physician clinical care and patient health: a review of systematic reviews**. *Int J Technol Assess Health Care* 2005;**21**:380-385.

29. Kaushal R, Shojania KG, Bates DW: **Effects of computerized physician order entry and clinical decision support systems on medication safety: a systematic review**. *Arch Intern Med* 2003;**163**:1409-1416.

30. Pearson SA, Moxey A, Robertson J, Hains I, Williamson M, Reeve J, Newby D: **Do computerised clinical decision support systems for prescribing change practice? A systematic review of the literature (1990-2007).** *BMC Health Serv Res* 2009;**9**:154.

31. Flodgren G, Pomey MP, Taber SA, Eccles MP: **Effectiveness of external inspection of compliance with standards in improving healthcare organisation behaviour, healthcare professional behaviour or patient outcomes.** *Cochrane Database Syst Rev* 2011;**11**:Cd008992.

32. Glasgow JM, Scott-Caziewell JR, Kaboli PJ: **Guiding inpatient quality improvement: a systematic review of Lean and Six Sigma**. *Jt Comm J Qual Patient Saf* 2010;**36**:533-540.

33. Alkhenizan A, Shaw C: **Impact of accreditation on the quality of healthcare services: a systematic review of the literature**. *Ann Saudi Med* 2011;**31**:407-416.

34. Greenfield D, Braithwaite J: **Health sector accreditation research: a systematic review.** *Int J Qual Health Care* 2008;**20**:172-183.

35. Main C, Moxham T, Wyatt JC, Kay J, Anderson R, Stein K: **Computerised decision support systems in order communication for diagnostic, screening or monitoring test ordering: systematic reviews of the effects and cost-effectiveness of systems.** *Health Technol Assess* 2010;**14**:1-227.

36. Veloski J, Boex JR, Grasberger MJ, Evans A, Wolfson DB: **Systematic review of the literature on assessment, feedback and physicians' clinical performance: BEME Guide No. 7.** *Medical teacher* 2006;**28**:117-128.

37. Ivers N, Jamtvedt G, Flottorp S, Young JM, Odgaard-Jensen J, French SD, O'Brien MA, Johansen M, Grimshaw J, Oxman AD: **Audit and feedback: effects on professional practice and healthcare outcomes**. *Cochrane Database Syst Rev* 2012;**6**:Cd000259.

38. Grimshaw JM, Thomas RE, MacLennan G, Fraser C, Ramsay CR, Vale L, Whitty P, Eccles MP, Matowe L, Shirran L, Wensing M, Dijkstra R, Donaldson C: **Effectiveness and efficiency of guideline dissemination and implementation strategies**. *Health Technol Assess* 2004;**8**:iii-iv,1-72.

39. Nicolay CR, Purkayastha S, Greenhalgh A, Benn J, Chaturvedi S, Phillips N, Darzi A: **Systematic review of the application of quality improvement methodologies from the manufacturing industry to surgical healthcare***. Br J Surg* 2012;**99**:324-335.

40. de Vos M, Graafmans W, Kooistra M, Meijboom B, Van Der Voort P, Westert G: **Using quality indicators to improve hospital care: a review of the literature**. *Int J Qual Health Care* 2009;**21**:119-129.

41. Weaver SJ, Lubomksi LH, Wilson RF, Pfoh ER, Martinez KA, Dy SM: **Promoting a culture of safety as a patient safety strategy: a systematic review**. *Ann Intern Med* 2013;**158**:369-374.

47. Shepperd S, Parkes J, McClaren J, Phillips C: **Discharge planning from hospital to home**. *Cochrane Database Syst Rev* 2004;**1**:Cd000313.

48. Ko HC, Turner TJ, Finnigan MA: **Systematic review of safety checklists for use by medical care teams in acute hospital settings--limited evidence of effectiveness**. *BMC Health Serv Res* 2011;**11**:211.

49. Damiani G, Pinnarelli L, Colosimo SC, Almiento R, Sicuro L, Galasso R, Sommella L, Ricciardi W: **The effectiveness of computerized clinical guidelines in the process of care: a systematic review**. *BMC Health Serv Res* 2010;**10**:2.

50. Aboelela SW, Stone PW, Larson EL: **Effectiveness of bundled behavioural interventions to control healthcare-associated infections: a systematic review of the literature**. *J Hosp Infect* 2007;**66**:101-108.

51. Stone PW, Pogorzelska M, Kunches L, Hirschhorn LR. **Hospital staffing and health care-associated infections: a systematic review of the literature**. *Clin Infect Dis* 2008;**47**:937-344.

52. Ketelaar NA, Faber MJ, Flottorp S, Rygh LH, Deane KH, Eccles MP: **Public release of performance data in changing the behaviour of healthcare consumers, professionals or organisations**. *Cochrane Database Syst Rev* 2011;**11**:Cd004538.

58. Lam-Antoniades M, Ratnapalan S, Tait G: **Electronic continuing education in the health professions: an update on evidence from RCTs**. *J Contin Educ Health Prof* 2009;**29**:44-51.

59. Thomson O'Brien MA, Freemantle N, Oxman AD, Wolf F, Davis DA, Herrin J: **Continuing education meetings and workshops: effects on professional practice and health care outcomes**. *Cochrane Database Syst Rev* 2001;**2**:Cd003030.

60. Parmelli E, Flodgren G, Beyer F, Baillie N, Schaafsma ME, Eccles MP: **The effectiveness of strategies to change organisational culture to improve healthcare performance: a systematic review**. *Implement Sci* 2011;**6**:33.

61. Scott T, Mannion R, Marshall M, Davies H: **Does organisational culture influence health care performance? A review of the evidence**. *J Health Serv Res Policy* 2003;**8**:105-117.

62. Brand CA, Barker AL, Morello RT, Vitale MR, Evans SM, Scott IA, Stoelwinder JU, Cameron PA: **A review of hospital characteristics associated with improved performance**. *Int J Qual Health Care* 2012;**24**:483-494.

63. Bright TJ, Wong A, Dhurjati R, Bristow E, Bastian L, Coeytaux RR, Samsa G, Hasselblad V, Williams JW, Musty MD, Wing L, Kendrick AS, Sanders GD, Lobach D: **Effect of clinical decision-support systems: a systematic review**. *Ann Intern Med* 2012;**157**:29-43.

64. Chaudhry B, Wang J, Wu S, Maglione M, Mojica W, Roth E, Morton SC, Shekelle PG: **Systematic review: impact of health information technology on quality, efficiency, and costs of medical care**. *Ann Intern Med* 2006;**144**:742-52.

65. Damiani G, Pinnarelli L, Scopelliti L, Sommella L, Ricciardi W: **A review on the impact of systematic safety processes for the control of error in medicine**. *Med Sci Monit* 2009;**15**:Ra157-66.

66. Hemens BJ, Holbrook A, Tonkin M, Mackay JA, Weise-Kelly L, Navarro T, Wilczynski NL, Haynes RB, CCDSS Systematic Review Team: **Computerized clinical decision support systems for drug prescribing and management: a decision-maker-researcher partnership systematic review**. *Implement Sci* 2011;**6**:89.

67. Jamal A, McKenzie K, Clark M: **The impact of health information technology on the quality of medical and health care: a systematic review**. *HIM J* 2009;**38**:26-37.

68. Kawamoto K, Houlihan CA, Balas EA, Lobach DF: **Improving clinical practice using clinical decision support systems: a systematic review of trials to identify features critical to success**. *BMJ* 2005;**330**:765.

69. Sahota N, Lloyd R, Ramakrishna A, Mackay JA, Prorok JC, Weise-Kelly L, Navarro T,

Wilczynski NL, Haynes RB, CCDSS Systematic Review Team: **Computerized clinical decision support systems for acute care management: a decision-maker-researcher partnership systematic review of effects on process of care and patient outcomes**. *Implement Sci* 2011;**6**:91.

70. Shojania KG, Jennings A, Mayhew A, Ramsay CR, Eccles MP, Grimshaw J: **The effects of on-screen, point of care computer reminders on processes and outcomes of care**. *Cochrane Database Syst Rev* 2009;**3**:Cd001096.

71. Wong K, Yu SK, Holbrook A: **A systematic review of medication safety outcomes related to drug interaction software**. *J Popul Ther Clin Pharmacol* 2010;**17**:e243-55

72. Grimshaw J, Eccles M, Thomas R, MacLennan G, Ramsay C, Fraser C, Vale L: **Toward evidence-based quality improvement. Evidence (and its limitations) of the effectiveness of guideline dissemination and implementation strategies 1966-1998**. *J Gen Intern Med* 2006;**21**(Suppl 2):S14-20.

73. Arora VM, Manjarrez E, Dressler DD, Basaviah P, Halasyamani L, Kripalani S: **Hospitalist handoffs: a systematic review and task force recommendations**. *J Hosp Med* 2009;**4**:433-440.

74. Gordon M, Findley R: **Educational interventions to improve handover in health care: a systematic review**. *Med Educ* 2011;**45**:1081-1089.

75. Mistiaen P, Francke AL, Poot E: **Interventions aimed at reducing problems in adult patients discharged from hospital to home: a systematic meta-review**. *BMC Health Serv Res* 2007;**7**:47.

76. Coulter A, Ellins J: **Effectiveness of strategies for informing, educating, and involving patients**. *BMJ* 2007;**335**:24-27.

77. Lewin SA, Skea ZC, Entwistle V, Zwarenstein M, Dick J: **Interventions for providers to promote a patient-centred approach in clinical consultations**. *Cochrane Database Syst Rev* 2001;**4**:Cd003267.

78. DelliFraine JL, Langabeer JR, 2nd, Nembhard IM: **Assessing the evidence of Six Sigma and Lean in the health care industry**. *Qual Manag Health Care* 2010;**19**:211-225.

79. Marshall MN, Shekelle PG, Leatherman S, Brook RH: **The public release of performance data: what do we expect to gain? A review of the evidence**. *JAMA* 2000;**283**:1866-1874.

80. Hysong SJ: **Meta-analysis: audit and feedback features impact effectiveness on care quality**. *Med Care* 2009;**47**:356-363.

81. Percarpio KB, Watts BV, Weeks WB: **The effectiveness of root cause analysis: what does the literature tell us?** *Jt Comm J Qual Patient Saf* 2008;**34**:391-398.

82. O'Brien MA, Rogers S, Jamtvedt G, Oxman AD, Odgaard-Jensen J, Kristoffersen DT, Forsetlund L, Bainbridge D, Freemantle N, Davis DA, Haynes RB, Harvey EL: **Educational outreach visits: effects on professional practice and health care outcomes.** *Cochrane Database Syst Rev* 2007;**4**:Cd000409.
